# Cinnamic acid induces apoptotic cell death and cytoskeleton disruption in human melanoma cells

**DOI:** 10.1186/1756-9966-32-31

**Published:** 2013-05-23

**Authors:** Evandro Luís de Oliveira Niero, Gláucia Maria Machado-Santelli

**Affiliations:** 1 Department of Cell and Developmental Biology, Institute of Biomedical Sciences, University of São Paulo, Av. Prof. Lineu Prestes, 1524, Cidade Universitária, 05508-000 São Paulo, SP, Brazil

**Keywords:** Cinnamic acid, Melanoma cells, Cytotoxicity, Apoptosis, Cytoskeleton, Micronuclei

## Abstract

Anticancer activities of cinnamic acid derivatives include induction of apoptosis by irreversible DNA damage leading to cell death. The present work aimed to compare the cytotoxic and genotoxic potential of cinnamic acid in human melanoma cell line (HT-144) and human melanocyte cell line derived from blue nevus (NGM). Viability assay showed that the IC_50_ for HT-144 cells was 2.4 mM, while NGM cells were more resistant to the treatment. The growth inhibition was probably associated with DNA damage leading to DNA synthesis inhibition, as shown by BrdU incorporation assay, induction of nuclear aberrations and then apoptosis. The frequency of cell death caused by cinnamic acid was higher in HT-144 cells. Activated-caspase 3 staining showed apoptosis after 24 hours of treatment with cinnamic acid 3.2 mM in HT-144 cells, but not in NGM. We observed microtubules disorganization after cinnamic acid exposure, but this event and cell death seem to be independent according to M30 and tubulin labeling. The frequency of micronucleated HT-144 cells was higher after treatment with cinnamic acid (0.4 and 3.2 mM) when compared to the controls. Cinnamic acid 3.2 mM also increased the frequency of micronucleated NGM cells indicating genotoxic activity of the compound, but the effects were milder. Binucleation and multinucleation counting showed similar results. We conclude that cinnamic acid has effective antiproliferative activity against melanoma cells. However, the increased frequency of micronucleation in NGM cells warrants the possibility of genotoxicity and needs further investigation.

## Introduction

Because there is no current effective treatment for metastatic melanoma and the average survival time is only 6 to 10 months [1,2], one way to control for malignancy is via prevention. In many cases, the term “prevention” is used to chemopreventive suppression or reversal of premalignant lesions even when the lesion is not completely eliminated [3,4]. Several studies have shown that the consumption of vegetables and fruits decreases the risk of many malignancies [5-7] and can protect against cancers [8-10]. Natural products have been well recognized as a source of drugs, and some plant extracts and compounds that are isolated from medicinal plants have been identified for their anti-cancer activities [11,12].

One anti-tumoral compound isolated from several plant-derived products is cinnamic acid. Cinnamic acid and its associated compounds can be found in coffee, apples, citric fruits, vegetable oils, propolis and wine. Cinnamic acid has a long history of human use as a component of plant-derived scents and flavoring agent [13]. Liu et al. [5] found that this compound induced tumor cell differentiation by modulating the expression of genes implicated in tumor metastasis and immunogenicity in cultured human melanoma cells.

Several researchers have also demonstrated the antioxidant activity of caffeic acid and its derivatives [14,15], which may be associated with cell death. Lee et al. [8] demonstrated that natural antioxidant compounds in diet, such as polyphenols in green tea, activate the MAPK pathway. Moreover, at high concentrations, these substances activate the caspase signaling cascade, which induces apoptosis in normal cells [8].

Lamartiniere et al. [16] showed that soy isoflavones such as genistein (another polyphenolic compound) act as chemopreventive agents against prostate and mammary cancers. One of the chemopreventive mechanisms against cancer is the induction of irreversible DNA damage, which results in cell death via apoptosis [17]. Impaired function of p53 increases the probability of proliferating cells with genetic abnormalities in some conditions [18,19]. This is due to the activation of p53 in response to unfavorable treatments, which results in genetic abnormalities such as DNA breakages [20,21], disruption of microtubules [22], lack of chromosome segregation at mitosis [23] or the incorrect termination of cell division, which can result in micronuclei formation [22].

The micronucleus test is widely used to detect chromosomal aberrations because micronuclei can originate from chromosomal fragments or disruptions in the mitotic spindle [24,25]. This assay has been used to evaluate the exposure levels of the human population to mutagenic or genotoxic agents [26-30] as well as in cell cultures to determine the mutagenic potential of drugs and/or natural compounds [31-33].

The screening of new compounds with anti-microbial and anti-inflammatory activities has resulted in the discovery of anti-tumor and chemopreventive properties of cinnamic acid and its derivatives [5,34-36]. Selective cytotoxicity in tumor cells is an important role to be analyzed to compare drug effects in cultured cells [37,38]. This study aimed to compare the cytotoxic and genotoxic potential of cinnamic acid in both a human melanocyte cell line of blue nevus and in cultured melanoma human cells.

## Materials and methods

### Cell cultures

HT-144 cell line, derived from malignant cutaneous melanoma, was obtained from American Type Culture Collection (ATCC). NGM cell line, derived from melanocytes of blue nevus, was obtained from Cell Bank of Rio de Janeiro (Brazil). All cultured cells were maintained in DMEM supplemented with 10% fetal bovine serum (FBS) at 37°C in a humidified atmosphere of 5% CO_2_. The experimental protocols were approved by the Ethics Committee of the Institute of Biomedical Sciences, University of São Paulo, Brazil (Protocol CEP-ICB n. 308/09).

### Cinnamic acid

Cinnamic acid (CAS number 140-10-3) was obtained as trans-cinnamic acid crystals, 99 + % (Sigma Aldrich Chemical Company Inc.) and the solutions were prepared by using 24 mg of the compound and 500 μL of ethanol. Phosphate buffered saline (PBSA) was added to complete 10 mL (final concentration at 16 mM). An appropriate control with DMEM, 20% PBSA and 1% ethanol was used.

### Cytotoxicity assay

The MTT kit (Promega) was used to evaluate the cytotoxicity. Briefly, 1 × 10^4^ cells were seeded in each well containing 100 μL of DMEM plus 10% of FBS in a 96-well plate. After 24 h, various concentrations of cinnamic acid were added. The control group received drug-free medium. After 2 days, 15 μL of “Dye Solution” were added to each well and the plates were incubated for additional 4 h. Then, 100 μL of “Solubilization/Stop Solution” were added in each well and the optical density was measured at 570 nm in an ELISA plate reader (BIO-RAD).

### Propidium iodide staining for flow cytometry

NGM and HT-144 cells (3 × 10^5^ cells/35 × 11 mm dishes) were incubated for 24 h and then treated with different concentrations of cinnamic acid. After 2 days the cells were harvested and submitted to fixation with 75% of ice-cold methanol at 4°C for 1 h. Cells were then washed with PBSA and suspended in propidium iodide staining solution containing 200 μL of PBSA, 20 μL of ribonuclease (10 mg/mL) and 20 μL of propidium iodide (10 μg/mL). The cell suspensions were incubated for 1 h at 4°C and 5,000 cells were analyzed by flow cytometry in each group (EasyCyte MINI - Guava Technologies).

### 5-bromo-2-deoxyuridine incorporation

After incubation and treatment with cinnamic acid the cells were submitted to BrdU (50 μM) (Sigma) incorporation for 30 minutes or 1 hour at 37°C. The samples were washed with PBSA and fixed with ethanol/acetic acid (3:1) for 15 minutes. The cells were incubated with HCl 2 M for 30 minutes. Then, we added antibody anti-BrdU (Sigma) (1:100) for 1 hour and, then, secondary antibody FITC-conjugated for 30 minutes.

The cells were treated with ribonuclease (10 mg/mL) and the nuclei were counterstained with propidium iodide (10 μg/mL). We analyzed 1,000 cells/coverslips.

### Activated-caspase 9 assay

NGM and HT-144 cells (3 × 10^5^ cells/35 × 11 mm dishes) were incubated for 24 h and subsequently treated with different concentrations of cinnamic acid. After 6, 12 or 24 hours the cells were harvested and suspended at 1 × 10^5^ cells/mL. Then, we added Caspase Reagent Working Solution (protocol by Guava Technologies) into the cell suspension. After incubation for 1 hour at 37°C we added 100 μL of 1× Apoptosis Wash Buffer in each sample and centrifuged them at 300 G for 7 minutes. The cells were resuspended in 200 μL of Caspase 7-AAD Working Solution. The samples were analyzed by using a flow cytometer (EasyCyte MINI - Guava Technologies).

### Immunoblots

The medium was removed after the treatments, and the cells were washed with PBSA and lysed with RIPA buffer [50 mM Tris–HCl (pH 7.5), 150 mM NaCl, 0.1% NP-40, 0.5% sodium deoxycholate, 1 mM EDTA and 2 mM EGTA]. The lysates were centrifuged and the supernatants were collected. 30 μg of protein were fractionated by SDS-PAGE on a 10% gel, and transferred to a PVDF membrane (Amersham Bioscience). A blocking solution (5% BSA (containing the phosphatase inhibitors NaF and orthovanadate)) was added to the membrane for 1 hour. The membrane was incubated overnight with an anti-p53 or anti-phospho-p53 (Ser15) (Abcam Inc.) antibodies diluted at 1:300. The immune complexes were detected by using the ECL Western blotting detection kit (Amersham Pharmacia). The ImageJ program was used for the densitometric analyses.

### M30, tubulin and actin staining

Cells were plated on coverslips (3 × 10^5^ cells/35 × 11 mm dishes). After 48 h of treatment, the cells were fixed with formaldehyde 3.7% for 30 minutes, washed with PBSA and treated with ribonuclease (10 mg/mL). To detect cytokeratin 18 fragments we added M30 antibody (FITC-conjugated) (CytoDEATH-Roche Labs) overnight at room temperature. The cells were submitted to immunofluorescence with anti-α and β-tubulin (Sigma, 1:200) overnight at room temperature and secondary antibody anti-mouse TRITC-conjugated.

In some cases, actin cytoskeleton was analyzed by using phalloidin FITC-conjugated and anti-α and β-tubulin with secondary antibody anti-mouse CY5-conjugated (Invitrogen, 1:200). Nuclei were counterstained with propidium iodide (10 μg/mL).

The images were analyzed by Laser Scanning Confocal Microscopy (Zeiss- LSM510) and we counted 1,000 cells/slide.

### Nuclear abnormalities frequency

Cells were plated on coverslips (3 × 10^5^ cells/35 × 11 mm dishes), grown for 24 h and treated with cinnamic acid at different concentrations. After 48 h of treatment, the cells were fixed with formaldehyde 3.7% for 30 minutes, treated with ribonuclease (10 mg/mL) for 30 minutes and stained with propidium iodide (10 μg/mL) during 20 minutes. We analyzed 2,000 cells/coverslips and the nuclear aberrations (micronucleation, binucleation and multinucleation) were counted according to the classification of Tolbert et al. [39], modified by Manelli-Oliveira and Machado-Santelli [40].

### Statistics

Statistical analysis on cell viability was achieved by χ^2^ tests to determine a statistical difference between the treated cells and the control group for each concentration. Flow cytometry, BrdU incorporation, protein expression, M30 labeling and nuclear aberrations data were analyzed by using the two way ANOVA test to verify a possible concentration-response or time-response relationship. We also analyzed cell death by using Multidimensional Nonlinear Descriptive Analysis (estimation by using negative binomial model).

## Results

### Cell viability

A wide range of concentrations of cinnamic acid (0.0125 to 3.2 mM) was used to test the cytotoxic effects of the compound on blue nevus-derived melanocytes and melanoma-derived cells. The MTT cell viability assay showed an IC_50_ of 2.4 mM in HT-144 cells. Thus, all of the experiments were performed using two cinnamic acid concentrations: 0.4 mM and 3.2 mM, which are below and above the IC_50_, respectively. The NGM cell line was more resistant to the treatment. The IC_50_ in the NGM cells was not reached (even at 3.2 mM cinnamic acid), and the cell growth was very similar among the different treatment groups compared to the control cells.

We did not observe differences between the control using 1% ethanol and the control using only free medium. Other experiments repeated this result. So, from here on, we will mention only the control with free medium.

### Cell cycle analysis

The effect of cinnamic acid on cell viability may be a result of cell cycle phase-specific arrest or cell death induction. DNA quantification was performed using flow cytometry and showed a decreased percentage in S phase in HT-144 cells treated with 3.2 mM cinnamic acid (16.08% to 6.35%) and an increased frequency of hypodiploid cells after treatment with the same concentration (from 13.80% in the control group to 25.78% in the 3.2 mM group) (Table 1). These data showed that the drug, at the highest concentration, induced cell death in HT-144 cells and decreased the percentage of cells in S phase.

**Table 1:** Effect of cinnamic acid on cell cycle of HT-144 and NGM cells after 48 h exposure

**Cell line**	**Cell cycle phases**	**Control groups**	**Treated groups**	
			**0.4 mM**	**3.2 mM**
HT-144	Hypodiploid cells	13.80 ± 3.49	15.38 ± 0.86	25.78 ± 2.85^a^
	G0/G1 phases	42.90 ± 4.37	45.12 ± 2.32	47.99 ± 5.30
	S phase	16.08 ± 2,49	12.22 ± 2.01	6.35 ± 1.21^b^
	G2/M phases	18.69 ± 4.10	19.95 ± 1.95	15.07 ± 2.04
	Polyploid cells	9.16 ± 3.14	7.80 ± 2.43	5.19 ± 1.84
NGM	Hypodiploid cells	11.25 ± 3.88	8.51 ± 3.10	43.31 ± 5.46^b^
	G0/G1 phases	64.81 ± 3.43	64.72 ± 7.43	40.46 ± 3.94^b^
	S phase	5.59 ± 1.56	4.48 ± 1.43	2.24 ± 1.01
	G2/M phases	13.67 ± 1.43	16.82 ± 2.36	10.93 ± 3.65
	Polyploid cells	4.93 ± 1.45	5.70 ± 1.27	3.21 ± 1.46

The numbers represent the frequency of cells (%) in each phase of the cell cycle according to DNA quantification by flow cytometry. Results are showed as Mean ± SD.

^a^ Significantly different (p≤0.01) from control group and 0.4 mM treated group.

^b^ Significantly different (p≤0.05) from control group.

NGM cells showed few differences compared to the melanoma cells. We did not observe a significant reduction in the percentage of cells in S phase. In contrast, NGM cells showed a decreased percentage of cells in G0/G1 after treatment with 3.2 mM cinnamic acid (from 64.81% in the control group to 40.46% in the treated group). We also detected changes in the percentage of hypodiploid cells (11.25% in the control group and 43.31% in the group treated with 3.2 mM of the drug).

**Figure 1:**
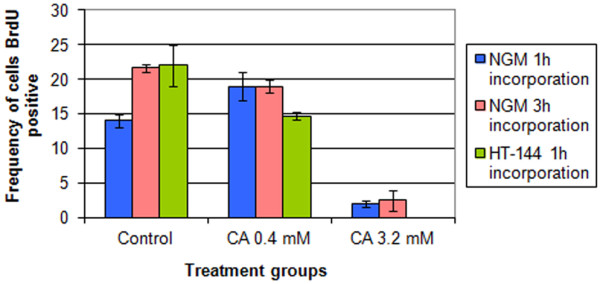
**BrdU incorporation in NGM and HT-144 cells treated with cinnamic acid.** The cells incorporate BrdU for different periods after 48 hours of treatment with two concentrations of cinnamic acid. We observed significative effects of cinnamic acid on DNA synthesis only in cells treated with 3.2 mM of the drug. Bars = standard error.

S-phase was further analyzed in the BrdU incorporation experiments. The incorporation time periods were 1 h and 3 h in NGM cells and 1 h in HT144. A time interval of 3 hours was tested in the NGM cells because of their slower proliferation rate (data obtained by growth curves). In addition, the BrdU incorporation experiments showed a significant reduction in the percentages of cells in S phase in both cell lines after treatment with 3.2 mM cinnamic acid (Figure 1). However, we found no differences between the periods of incorporation (Figure 1). The reduction in the percentage of cells in S phase was more significant in HT-144 cells than in NGM cells. In these cells, the BrdU incorporation index decreased from 22% in the control group to 0% in the group treated with 3.2 mM cinnamic acid (Figure 1).

We also used a 0.05 mM cinnamic acid concentration along the study; however we did not find changes in comparison to the control group.

### Cell death detection

The interference of cinnamic acid in the cell cycle may result in cell death. To confirm this hypothesis, the cells were labeled with M30. The HT-144 cell line showed an increased frequency in labeled cells after 24 h of treatment with both concentrations of the drug and this increase was time-dependent (Table 2).

**Table 2:** Frequency of HT-144 cells positive for M30 (%) after treatment with cinnamic acid

**Time of treatment**	**Control**	**0.4 mM**	**3.2 mM**
24 hours	0.80 ± 0.07	5.00 ± 0.09^a^	7.30 ± 1.02^a^
48 hours	1.20 ± 0.06	12.30 ± 1.95^a^	27.03 ± 2.36^a^

Results are showed as Mean ± SD.

^a^ Significantly different (p≤0.05) vs control group.

**Figure 2:**
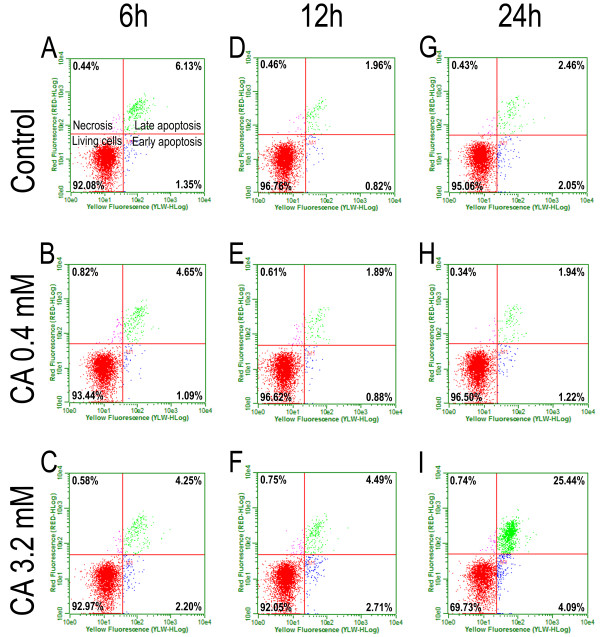
**Activated-caspase 9 assay to cell death analysis on HT-144 cells.** The activated-caspase 9 kit (GE Healthcare) was used to detect different stages of cell death. The cells were treated at 0.4 or 3.2 mM cinnamic acid for 6 (**A**, **B**, **C**), 12 (**D**, **E**,**F**) and 24 hours (**G**, **H**, **I**). We can observe increased frequency of apoptotic cells after 24 h of treatment at 3.2 mM cinnamic acid.

**Figure 3:**
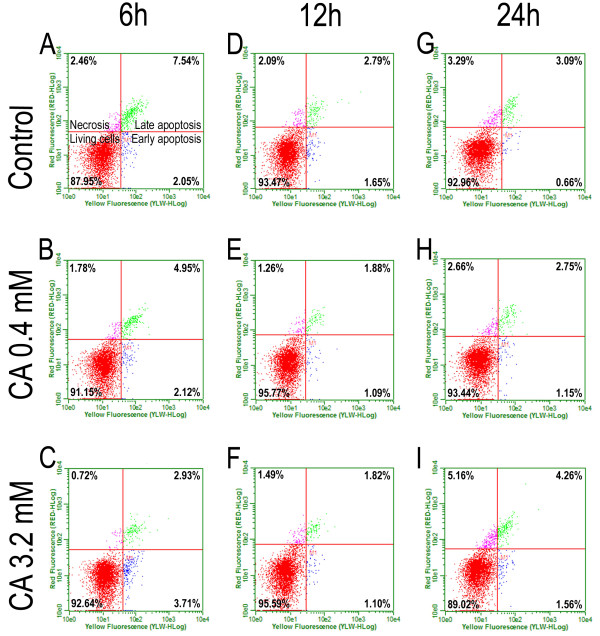
**Activated-caspase 9 assay to cell death analysis on NGM cells.** The activated-caspase 9 kit (GE Healthcare) was used to detect different stages of cell death. The cells were treated at 0.4 or 3.2 mM cinnamic acid for 6 (**A**, **B**, **C**), 12 (**D**, **E**, **F**) and 24 hours (**G**, **H**, **I**). The results did not show differences among the control groups and the treated groups.

The activated-caspase 9 assay confirmed the data obtained from the M30 labeling of HT-144 cells (Figure 2). Because we could not analyze the cell death in the NGM cell line using M30 labeling, we performed the active-caspase 9 assay in NGM cells (Figure 3) to compare the effects of cinnamic acid in both cell lines. Cells exposed to ultraviolet radiation for 1 minute were used as a positive control. This experiment verified that both cell lines could functionally activate the caspase cascade during the cell death process.

We did not observe significant differences between the control and treated groups after 6 or 12 hours of drug exposure (Table 3). Interestingly, the apoptotic cascade in the HT-144 cells was initiated approximately 24 hours after treatment with 3.2 mM cinnamic acid, specifically, when the frequency of cell death changed from 5% in the control group to 30% in the treated group. Our results indicated that there was no significant increase in apoptotic cell frequency after treatment with 0.4 mM of the drug.

**Table 3:** Frequencies (%) of apoptotic cells (early + late apoptosis) in HT-144 and NGM cell lines after treatment with cinnamic acid in different times and concentrations

**Cell line**	**Time of treatment**	**Control groups**	**Treated groups**		
			**0.05 mM**	**0.4 mM**	**3.2 mM**
HT-144	6 hours	7.48	6.96	5.74	6.45
	12 hours	2.78	2.29	2.77	7.20
	24 hours	4.51	4.52	3.16	29.53^a^
NGM	6 hours	9.59	8.83	7.07	6.64
	12 hours	4.44	4.46	2.97	2.92
	24 hours	3.75	4.64	3.90	5.82

The results were obtained by quantification of cells positive to activated-caspase 09 by using a flow cytometer.

^a^ Significantly different from control group according to Multidimensional Nonlinear Descriptive Analysis.

Furthermore, there were no differences between the control and treated groups of NGM cells after 24 hours of treatment with cinnamic acid (Table 3). The frequency of apoptotic cells in the control group was approximately 5%, and the frequency of apoptosis in the NGM cell line did not reach 9% in any group. The statistics confirmed that the differences observed were not significant.

**Figure 4:**
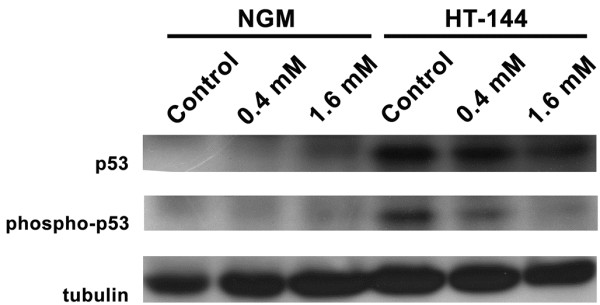
**p53 and phospho-p53 levels in NGM and HT-144 cells after cinnamic acid exposure for 24 hours.** There were no differences in p53 or phospho-p53 levels after treatment of NGM cells. HT-144 cells showed decreased level of p53 and phospho-p53 after treatment with cinnamic acid. Tubulin was used as a loading control.

The western blotting analysis showed that both cell lines express the p53 protein. We could not confirm the selective effects of cinnamic acid by the total p53 quantification or p53 phosphorylation because apoptosis in HT-144 cells was not directly associated with the increase of p53 expression or phosphorylation (Figure 4).

### Cell morphology

**Figure 5:**
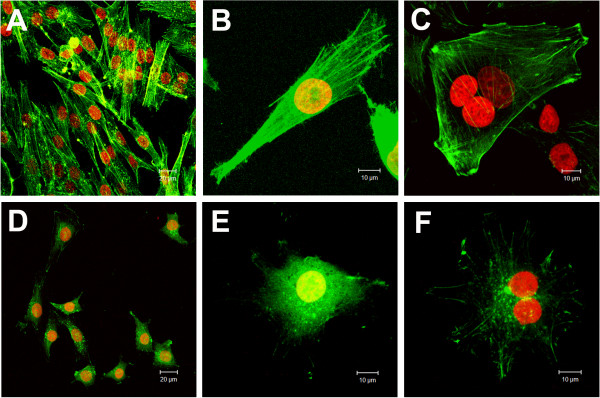
**Effects of cinnamic acid on microfilaments organization of HT-144 cells.** Images obtained by Laser Scanning Confocal Microscopy of phalloidin FITC-conjugated staining (green) preparations: **A,B,C)** HT-144 control cells; **D,E,F)** HT-144 cells treated with 3.2 mM cinnamic acid. DNA was counterstained with propidium iodide (red). Note the stress fiber formation in control cells (above) and the decreasing of peripheral actin filaments and perinuclear accumulation of F-actin in treated groups (below).

**Figure 6:**
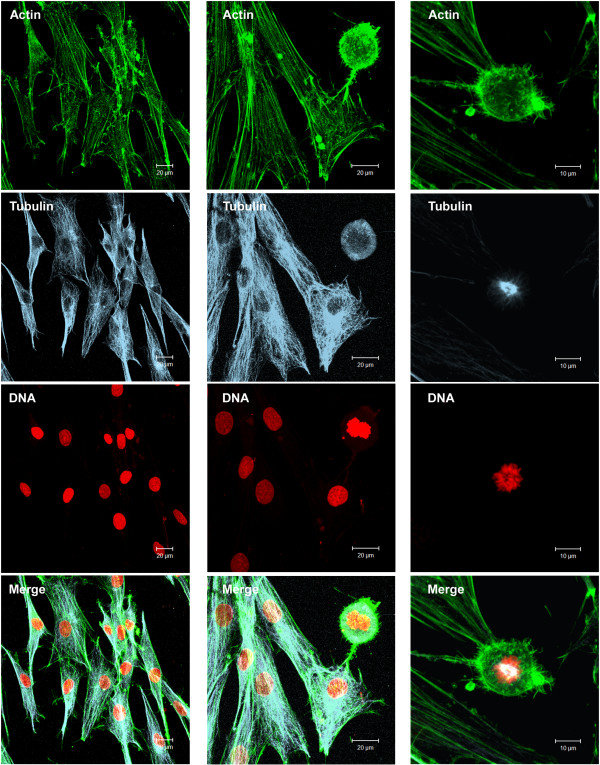
**Cytoskeleton organization in NGM control cells.** F-actin (green) was stained with phalloidin FITC-conjugated. Microtubules (blue) were labeled with anti-α and β tubulin and secondary antibody CY-5-conjugated. DNA was counterstained with propidium iodide (red). Note the stress fiber formation (actin filaments). The cells showed a microtubule network that was very finely departed from the centrosome region near the nucleus. We can also observe a mitotic cell (right column). The images were obtained by Laser Scanning Confocal Microscopy.

The morphological changes observed using microscopy after treatment with cinnamic acid and the BrdU incorporation data suggested that the drug targets the cell cycle. Thus, we analyzed the cytoskeleton of the cells after drug treatment. The control groups of both cell lines commonly appeared as fusiform cells, with microfilaments that formed parallel stress fibers (Figures 5A-C, 6). After treatment with 0.4 mM cinnamic acid, the HT-144 cells showed a triangular or stellate morphology, and an altered orientation of actin filaments. The microfilament disorganization was higher in the melanoma cells after treatment with 3.2 mM of the drug (Figure 5D-F). We detected important decrease in the microfilament density in the peripheral cytoplasm and an accumulation of fragmented F-actin near the nucleus in HT-144 cells treated with the higher drug concentration.

**Figure 7:**
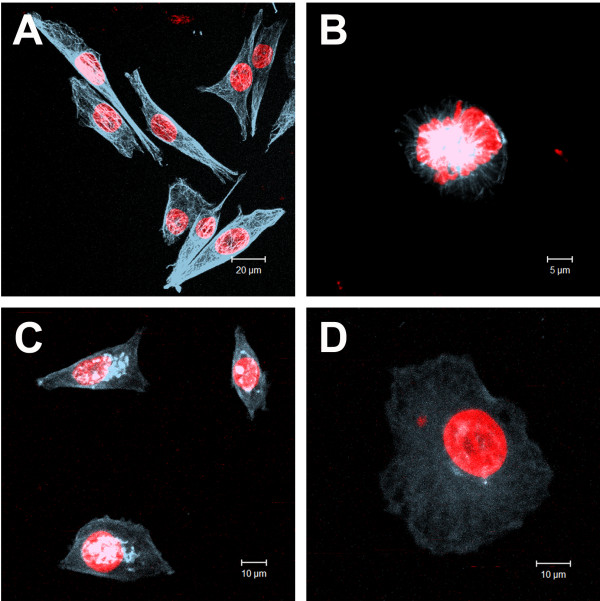
**Effects of cinnamic acid on microtubules organization of HT-144 cells.** Images obtained by Laser Scanning Confocal Microscopy of anti-tubulin immunofluorescence (blue) preparations: **A)** interphasic HT-144 control cells; **B)** mitotic HT-144 control cell; **C,D)** HT-144 cells treated with 3.2 mM cinnamic acid. DNA was counterstained with propidium iodide (red). We can observe cells with a microtubule network that was very finely departed from the centrosome region near the nucleus (up left) and a normal mitosis (up right). On the other hand, we found cells with microtubule disorganization and tubulin bunches near the nuclei.

We also observed microtubule disruption in HT-144 cells after treatment with cinnamic acid. Cells treated with 0.4 mM cinnamic acid maintained a normal distribution of microtubules, whereas treatment with 3.2 mM induced very diffuse labeling in the cytoplasm with accumulation around the cell nuclei (Figure 7).

**Figure 8:**
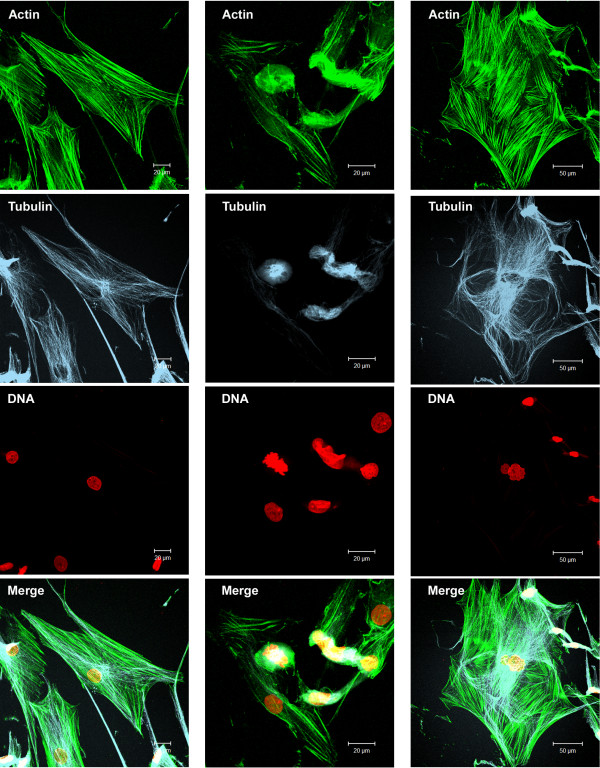
**Cytoskeleton organization in NGM cells treated with 3.2 mM cinnamic acid.** The cells were treated with the drug for 48 hours. F-actin (green) was stained with phalloidin FITC-conjugated. Microtubules (blue) were labeled with anti-α and β tubulin and secondary antibody CY-5-conjugated. DNA was counterstained with propidium iodide (red). The images were obtained by Laser Scanning Confocal Microscopy. Note that there are cells with normal cytoskeletal organization (left column) and cells with drastic morphological changes (intermediate and right columns).

Treatment with 3.2 mM cinnamic acid induced robust morphological changes in some NGM cells. In addition to changes that occurred in less than 2% of the cases, a cytoskeletal analysis revealed the presence of coiled actin filaments and microtubules (Figure 8). Moreover, the nuclei exhibited an alteration in their morphology, which were observed in NGM cells that were treated with 3.2 mM cinnamic acid; however, a low frequency was observed when compared to HT-144 cells. There was no cytoskeleton reorganization in the NGM cells treated with 0.4 mM of the drug.

**Figure 9:**
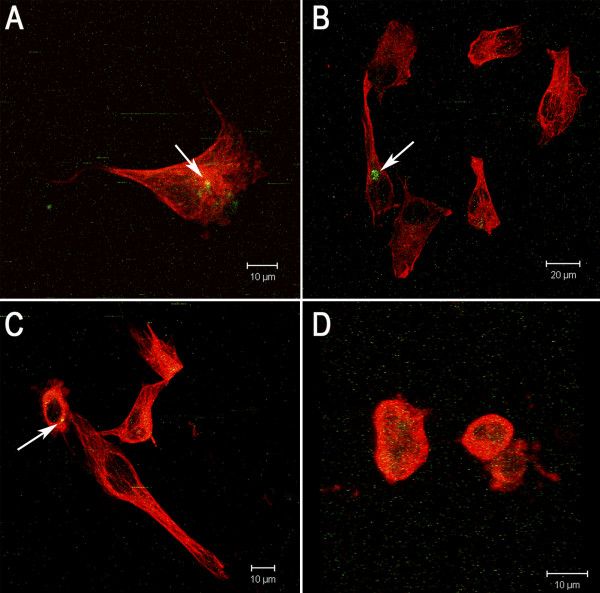
**M30 and tubulin labeling in HT-144 cells.** HT-144 cells were treated with 0.4 or 3.2 mM cinnamic acid for 24 or 48 hours. Fragmented cytokeratin 18 (green) were labeled with M30 antibody FITC and microtubules (blue) were labeled with anti-α and β tubulin and secondary antibody TRITC-conjugated. **A,B)** cells with intact microtubules and M30(+); **C)** cells with microtubule disruption and M30(+); **D)** cells with microtubule disruption and M30(–). Arrows = M30 staining. The results demonstrate that cell death and microtubule disorganization are independent events in our system. The images were obtained by Laser Scanning Confocal Microscopy.

To determine if there was an association between the morphological changes and apoptosis, we subjected the HT-144 cells to M30 and tubulin labeling simultaneously. The cells exhibited intact microtubules and M30(+) (Figure 9A-B), microtubule disruption and M30(+) (Figure 9C) and microtubule disruption and M30(–) (Figure 9D). Thus, the apoptotic process and microtubule disorganization are independent events in this model system.

### Nuclear aberrations

Because changes in apoptotic frequencies could be caused by direct DNA breakage or chromosomal loss due to microtubule disruption, we searched for cells with nuclear alterations to evaluate the genotoxic potential of cinnamic acid and analyzed the micronuclei frequency in HT-144 and NGM cells. The HT-144 control group showed 1.97% micronucleated cells. Both cinnamic acid concentrations increased the frequencies of the micronucleated cells: 3.13% with 0.4 mM and 6.07% with 3.2 mM cinnamic acid (Table 4).

**Table 4:** Effect of cinnamic acid on formation of nuclear aberrations in NGM and HT-144 cells after 48 h exposure

**Cell line**	**Group**	**Micronucleated cells**	**Cells with nuclear buds**	**Binucleated cells**	**Multinucleated cells**
HT-144	Control	1.97 ± 0.04	0.20 ± 0.05	1.83 ± 0.02	0.43 ± 0.06
	0.05 mM	2.01 ± 0.06	0.24 ± 0.06	1.79 ± 0.04	0.52 ± 0.03
	0.40 mM	3.13 ± 1.03^a^	0.40 ± 0.02	4.23 ± 1.03^a^	0.67 ± 0.04
	3.20 mM	6.07 ± 1.45^b^	1.30 ± 0.02^b^	5.87 ± 0.98^a^	1.17 ± 0.12^a^
NGM	Control	1.38 ± 0.06	0.15 ± 0.01	0.20 ± 0.03	0.05 ± 0.02
	0.05 mM	1.27 ± 0.04	0.19 ± 0.04	0.29 ± 0.02	0.25 ± 0.08
	0.40 mM	1.15 ± 0.01	0.10 ± 0.03	0.37 ± 0.07	0.00 ± 0.00
	3.20 mM	3.07 ± 0.03^a^	0.44 ± 0.02^a^	0.53 ± 0.06	0.00 ± 0.00

The numbers represent the frequency of cells (%) with nuclear alterations. Results are showed as Mean ± SD.

^a^ Significantly higher (p ≤ 0.05) than control group.

^b^ Significantly higher (p ≤ 0.05) than control group, group treated with 0.05 mM and group treated with 0.4 mM cinnamic acid.

The frequencies of the binucleated cells also increased when the cells were treated with both drug concentrations: from 1.83% in the control cells to 4.23% and 5.87% after treatment with 0.4 mM and 3.2 mM cinnamic acid, respectively. The frequency of cells with nuclear buds and multinucleated cells were also higher in the treated group compared to the control group; however, the effects were milder, and a significant difference was observed in only the group treated with 3.2 mM cinnamic acid. The frequency of cells with nuclear buds increased from 0.2% to 1.3% in the control group after treatment. Moreover, the presence of multinucleated cells increased from 0.43% to 1.17% in the control group after treatment.

NGM cells also showed an increased frequency in the presence of cells with micronuclei and/or nuclear buds after treatment with cinnamic acid. However, our results demonstrated milder effects in this cell line (Table 4). The control group showed a basal rate of micronucleated cells of 1.38%, while the group treated with 3.2 mM cinnamic acid exhibited an increase in frequency to 3.07%. However, we could not detect alterations using other concentrations. The frequency of cells with nuclear buds was also higher after treatment with 3.2 mM cinnamic acid (0.15% in the control group and 0.44% in the treated group); however, this was not observed when using other concentrations.

## Discussion

The decreasing effect of cinnamic acid on HT-144 cell viability was consistent with previous studies. Liu et al. [5] found that cinnamic acid reduced cell proliferation of glioblastoma, melanoma, prostate and lung carcinoma cells by 50% at concentrations between 1.0 and 4.5 mM. Using a different drug treatment regime, Ekmekcioglu et al. [41] showed that the IC_50_ of cinnamic acid was between 4.0 and 5.0 mM in Caco-2 cells.

Previous *in vivo* studies indicated that acute lethal doses (LD_50_) of cinnamic acid was achieved at 160-220 mg/kg (ip) in mice, 2.5 g/kg (oral) in rats and 5 g/kg (dermal) in rabbits. Thus, cinnamic acid exhibits a low toxicity [42]. Other studies have shown that caffeic acid phenethyl ester (cinnamic acid-derivative) exhibits a cytotoxic activity in different oral carcinoma cells [43] and that cinnamic acid protects DNA against fragmentation caused by hydrogen peroxide in V79 cells [44].

We could not determine the IC_50_ in NGM cells, despite treatment with the highest drug concentration (3.2 mM). Because cinnamic acid showed preferential activity against cancer cells, it is important to identify safe drug concentrations for use *in vivo* against cancer. The IC_50_ value can change according to the cell type, and it can reach 20.0 mM in fibroblasts [5]. This variation may be related to the cell type. Lee et al. [8] demonstrated that dietary compounds with antioxidant properties, such as polyphenols in green tea, can activate the MAPK pathway. They suggested that the tumor suppressor protein p53 and p38 MAPK are involved in the apoptotic process of tumor cells. Nevertheless, these substances, when used at high concentrations, can activate the caspase cascade and induces apoptosis in normal cells [8]. Thus, it is important to comprehend the action of these drugs at different concentrations in different systems to confirm its preferential activity against a target cell type.

Drugs that cause DNA breakage commonly result in cell cycle arrest and the activation of apoptosis [40]. Several of these drugs cause nuclear alterations by disruption of cytoskeletal organization. Microtubule disruption could also cause G2/M arrest prior to inducing cell death by apoptosis [45,46]. Thus, we investigated the cytoskeletal patterns of cells that were treated with cinnamic acid.

The control group showed a microtubule network that was very finely departed from the centrosome region near the nucleus. A visible disorganization of the tubulin filaments was detected in interphasic treated cells. Cells treated with 3.2 mM cinnamic acid showed diffuse cytoplasmic staining and protein accumulation around the nucleus. Cells treated with a 0.4 mM dose of the drug did not demonstrate alterations in the organization of their microtubule cytoskeleton.

Cytoplasmic retraction [47,48] is a characteristic of apoptosis, and cytoskeletal disorders have been implicated in this process [49]. Actin cleavage has been associated with many characteristics of pre-apoptotic cells [50], and microfilament reorganization is essential to apoptotic body formation in later stages of cell death [47].

The morphological changes observed in these cells revealed an association with actin filament depolymerization. Similar effects were shown in studies conducted by Boggio et al. [51], which demonstrated that human fibroblasts from keloids treated with verapamil, a calcium antagonist, showed an altered bipolar to spherical morphology. Boggio et al. [51] showed disassembly of the actin network with the formation of shorter stress fibers in fibroblasts treated with verapamil. This was strongly associated with a change in cell morphology.

The treatment of cells using anti-mitotic agents, such as taxol and taxotere, which maintain tubulin polymerization, revealed interesting alterations in the actin cytoskeleton. In these studies, MCF7 cells were treated with taxol or taxotere at concentrations of 10 μM or higher, which resulted in a decrease in peripheral microfilaments and progressive cytoplasmic actin accumulation and actin rings around the nuclei [52]. We demonstrated that the effects of cinnamic acid on the actin cytoskeleton in our model system were similar to those observed in other systems using different drugs. Cells treated with 3.2 mM cinnamic acid showed a sharp reduction in peripheral microfilaments, which was in contrast with many strongly stained clusters of F-actin located around the nuclei.

Cytoskeletal damage is a characteristic of pre-apoptotic cells [50]. Mills et al. [53] demonstrated cytoskeletal alterations during apoptosis and suggested a rearrangement of the peripheral actin ring in the cell. During bleb formation, actin and myosin filaments slide over each other, resulting in contraction of the cell border toward the center. This process impairs the binding of actin filaments to the cell membrane.

The mechanism by which cinnamic acid causes microfilament disorganization is not well understood; however, because taxol does not exhibit direct effects on microfilaments, this suggests interdependency between actin filaments and microtubules [52].

The disorganization of microtubules in cells treated with cinnamic acid may be directly caused by impairment in the tubulin molecules or indirectly by an alteration in the molecules associated with microtubule polymerization. It is known that the dynamic equilibrium of tubulin may be altered at high concentrations of free cytosolic calcium (higher than 10^-7^ M), which results in the depolymerization of microtubules [54].

Studies using other natural compounds have shown that the induction of cell death by caffeic acid and curcumin in HL-60 cells [8] and L929 mouse fibroblasts (Thayyllathil at al., 2008), respectively, is associated with mitochondrial disruption, which may be due to an augmented concentration of calcium that results in cytoskeletal disruption. These results are similar to the observations found in our system.

Our results allow us to affirm that microtubule depolymerization, as well as microfilament disorganization, occurred after exposure to 3.2 mM cinnamic acid. Microtubule disruptions have been previously described as a trigger of the apoptotic pathway, which eventually results in cell death [54].

Our data suggest that there is no relationship between the effects of cinnamic acid on cytoskeletal elements and apoptotic induction. We have demonstrated that M30 staining and microtubule disorganization are, at least in part, independent events.

Caffeic acid, another cinnamamide compound, causes apoptosis in HL-60 cells via mitochondrial dysfunction [8]. Previous studies have shown a relationship between cancer chemotherapeutic agents targeting microtubules and apoptosis [55,56]. The flow cytometry assay did not show G2/M arrest; however, microtubule disorganization was caused by cinnamic acid treatment. Thus, the apoptotic events observed in our study were not caused by cytoskeletal reorganization. Tseng et al. [57] studied podophylotoxin and suggested that mitotic arrest is not a prerequisite for apoptosis, although they often can occur concomitantly.

The present data suggest that microtubule disorganization after cinnamic acid exposure is dependent on the drug concentration. In our system, cytoskeletal disorganization is mainly responsible for the formation of nuclear aberrations.

We clearly observed apoptotic HT-144 cells, as assessed by phosphorylated cytokeratin 18. The M30 antibody stains cells in early apoptosis. In the present study, we showed that the apoptotic process initiates after 24 hours of treatment and that these effects were dependent on drug concentration, indicating that longer treatments with cinnamic acid could elicit a more robust response in cell viability.

M30 staining was not observed in NGM cells independent of the treatment. Cytokeratin 18 is usually found in the epithelial cells and is not expressed in normal melanocytes; however, some studies have associated its presence in melanoma cells with a worse prognosis [58,59]. The HT-144 cells were positive for phospho-cytokeratin 18 after treatment with cinnamic acid. These data further characterize the HT-144 cell line and show significant differences between the cell lines, providing new information regarding the HT-144 cell line.

Quantification of picnotic and fragmented nuclei showed that less than 1% of cells were apoptotic cells (data not shown). This could occur because many apoptotic cells are in suspension. Thus, we used flow cytometry to ensure that all of the cells would be quantified. The annexin-V assay did not reveal any differences among the groups of cells, except in groups of cells that were treated for long time periods. This result allowed us to infer that phosphatidylserine could not be exposed in our system during early cell death.

Caspase 9 is an initiator caspase that is usually associated with the activation of effector caspases, including caspase 3 and caspase 7 [60,61]. The activation of caspase 9 confirmed the results obtained by M30 staining in HT-144 cells and showed that cell apoptosis was induced after 24 hours of treatment with cinnamic acid. NGM cells were resistant to the treatment.

Several studies have demonstrated the antioxidant activity of similar compounds such as caffeic acid and derivatives [14,15]. This antioxidant activity was associated with the induction of the cell death process according to Lee et al. [8]. This authors showed that treatment with caffeic acid activated the MAPK cascade, including p38 MAPK, which phosphorylated p53 [62,63] in the human leukemia cell line HL-60. However, contrary to other malignancies, studies have failed to associate anticancer potential of some agents with p53 activity in melanoma, and our results showed decreased p53 expression and phosphorylation in HT-144 cells treated with cinnamic acid. So, we could not establish a relation between apoptosis and p53 phosphorylation in our system.

Many natural compounds with cytotoxic activity can cause nuclear alterations by disrupting cell separation during mitotic process. These disruptions result in the initiation of an aneugenic pathway [32,33,64]. According to Efthimiou et al. [33], the aneugenic potential is one event that can result in the carcinogenic process. Thus, an important aspect to be evaluated in the study of natural products is their genotoxic potential.

Chen et al. [65] showed that micronuclei may be produced by chromosomal breakage and/or whole chromosomal loss. In our studies, even at 0.4 mM cinnamic acid, an increase in the frequency of micronucleated cells was observed. The higher frequencies of micronucleated cells in NGM and HT-144 cells treated with cinnamic acid revealed genotoxic activity.

Treatment with cinnamic acid efficiently decreased HT-144 melanoma cell viability in culture at a concentration of 3.2 mM. Our study demonstrates that the antiproliferative activity of the drug is associated with caspase 9 activation, but not p53 phosphorylation, after 24 h treatment. We showed that HT-144 cells presented phospho-cytokeratin 18 and that the M30 staining was efficient in detecting early apoptosis in this cell line. Cinnamic acid showed genotoxic potential at both tested concentrations, inducing the formation of micronucleated cells. This activity was, at least in part, a consequence of cytoskeletal disorganization. Thus, despite the genotoxic effects observed, the anti-proliferative activity of cinnamic acid at a concentration of 3.2 mM in melanoma cells suggests its potential use as an adjuvant in melanoma therapy.

## Competing interests

The authors declare that they have no competing interests.

## Authors’ contributions

ELON and GMMS defined the research theme, designed methods and experiments, analyzed the data and critically read, revised and approved the final manuscript. ELON carried out the laboratory experiments.
